# Insights Into the Use of a Digital Healthy Aging Coach (AGATHA) for Older Adults From Malaysia: App Engagement, Usability, and Impact Study

**DOI:** 10.2196/54101

**Published:** 2024-05-21

**Authors:** Pei-Lee Teh, Andrei O J Kwok, Wing Loong Cheong, Shaun Lee

**Affiliations:** 1 School of Business Monash University Malaysia Subang Jaya Malaysia; 2 School of Pharmacy Monash University Malaysia Subang Jaya Malaysia

**Keywords:** digital health, older adults, digital divide, aging, pilot, Malaysia, coach, digital access, social barrier, virtual, virtual coach, digital tool, tool, engagement, gamification, user experience, app, technology tool, digital literacy, user experience design, decision support, support

## Abstract

**Background:**

Digital inclusion is considered a pivotal social determinant of health, particularly for older adults who may face significant barriers to digital access due to physical, sensory, and social limitations. Avatar for Global Access to Technology for Healthy Aging (AGATHA) is a virtual healthy aging coach developed by the World Health Organization to address these challenges. Designed as a comprehensive virtual coach, AGATHA comprises a gamified platform that covers multiple health-related topics and modules aimed at fostering user engagement and promoting healthy aging.

**Objective:**

The aim of this study was to explore the perception and user experience of Malaysian older adults in their interactions with the AGATHA app and its avatar. The focus of this study was to examine the engagement, usability, and educational impact of the app on health literacy and digital skills.

**Methods:**

We performed a qualitative study among adults 60 years and older from suburban and rural communities across six states in Malaysia. Participants were purposefully recruited to ensure representation across various socioeconomic and cultural backgrounds. Each participant attended a 1-hour training session to familiarize themselves with the interface and functionalities of AGATHA. Subsequently, all participants were required to engage with the AGATHA app two to three times per week for up to 2 weeks. Upon completion of this trial phase, an in-depth interview session was conducted to gather detailed feedback on their experiences.

**Results:**

Overall, the participants found AGATHA to be highly accessible and engaging. The content was reported to have a comprehensive structure and was delivered in an easily understandable and informative manner. Moreover, the participants found the app to be beneficial in enhancing their understanding pertaining to health-related issues in aging. Some key feedback gathered highlighted the need for increased interactive features that would allow for interaction with peers, better personalization of content tailored to the individual’s health condition, and improvement in the user-experience design to accommodate older users’ specific needs. Furthermore, enhancements in decision-support features within the app were suggested to better assist users in making health decisions.

**Conclusions:**

The prototype digital health coaching program AGATHA was well received as a user-friendly tool suitable for beginners, and was also perceived to be useful to enhance older adults’ digital literacy and confidence. The findings of this study offer important insights for designing other digital health tools and interventions targeting older adults, highlighting the importance of a user-centered design and personalization to improve the adoption of digital health solutions among older adults. This study also serves as a useful starting point for further development and refinement of digital health programs aimed at fostering an inclusive, supportive digital environment for older adults.

## Introduction

Digital inclusion is increasingly recognized as an essential social determinant of health and a necessary component of a healthy aging society [1]. Older adults risk being left behind from the digital transformation, as many societies rely on technology to access health information and services [2]. More importantly, older adults are more likely to be digitally excluded, especially if they experience physical and social barriers such as poor eyesight, hearing, and mobility, along with low technological literacy [3]. Without significant digital access and engagement improvements, ongoing technological developments and innovations may worsen inequalities for older adults. Existing studies have demonstrated that digital health coaching programs offering access to health information and services can augment health behaviors [4,5]. Given the lack of knowledge of how digital health coaching programs are adopted and utilized by older adults in developing economies, gaining insight into their perceptions and use of digital health technology is crucial to enable context-appropriate adoption of these programs [6].

Avatar for Global Access to Technology for Healthy Aging (AGATHA) is a virtual healthy aging coach that serves as a one-stop-shop platform for healthy aging promotion [7]. AGATHA has been developed by the World Health Organization (WHO) Regional Office of the Western Pacific and WHO Collaborating Center for Digital Health with the China Academy of Information and Communications Technology. The prototype app was developed in June 2021, comprising three key modules: exercise and falls, social care, and nutrition. The module contents were based on the WHO guidance on health-related topics and the Integrated Care for Older People (ICOPE) guidelines [8]. The ICOPE guidelines focus on six areas of intrinsic capacity: cognitive decline, limited mobility, visual impairment, malnutrition, hearing loss, and depressive symptoms [4,5]. To promote user engagement, AGATHA embeds gamification components such as scoring, progress tracking, and badges that can be shared with friends. AGATHA can be launched in English and Mandarin via any mobile or desktop web browser. The thinking behind developing AGATHA and its conceptualization through human-centered design approaches have been previously described [7]. The Institute on Aging at the University of the Philippines Manila, Pinetree Care Group in China, and Monash University Malaysia conducted early product evaluations involving older individuals as users of the tool and consumers of health information to guide the development of AGATHA. The evaluations encompassed a variety of methods, including observation and focus group interviews, to gain insights into the experiences and needs of this demographic.

Nevertheless, there is limited understanding on how digital technologies can be used to support and address specific health care challenges in many developing countries. Studies conducted in this field to date suggest that there are differences in digital literacy between developed and developing countries [9]. Therefore, the aim of this study was to explore Malaysian older adults’ perceptions and user experiences of the AGATHA app. The results of this study can be useful to provide insights into the health needs and interests of older adults. The results can further be used to optimize the healthy aging coach for long-term use and effectiveness as a health-promotion tool as well as to inform the development of other digital tools and programs targeting older adults in similar contexts.

## Methods

### Recruitment and Data Collection

The study was conducted between September 1, 2022, and October 18, 2022. Thirty older adults (aged ≥60 years) were recruited from the three major primary ethnicities (ie, Chinese, Indian, and Malay) in Malaysia, which collectively represent 90% of the population [10]. Older adults were selected across six states in Malaysia (Perak, Selangor, Kuala Lumpur, Penang, Johor, and Sarawak) to ensure representativeness. Purposive sampling is a widely used approach in qualitative studies to intentionally select informants based on their particular knowledge of the focus of empirical inquiry. This was considered to be an appropriate sampling method for this study as our selection criteria involved community-dwelling older adults 60 years and older from the six states with a large number of older adults. The recruitment was based on voluntary participation through purposive sampling using recruitment posters and invitations within the Monash University Gerontechnology Laboratory Volunteer Community, Malaysian Aging Research Network, volunteer networks among community centers and seniors’ associations (eg, Pusat Aktivity Warga Emas at Tanjung Malim, Perak), and online messenger platforms such as WhatsApp messenger.

### Research Design

**Figure 1:**
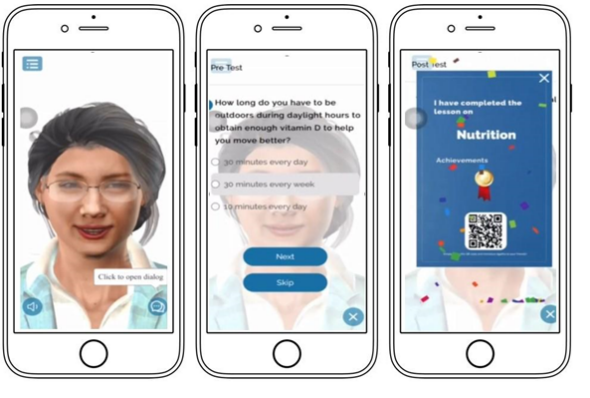
Screenshots of the Avatar for Global Access to Technology for Healthy Aging (AGATHA) app.

**Figure 2:**
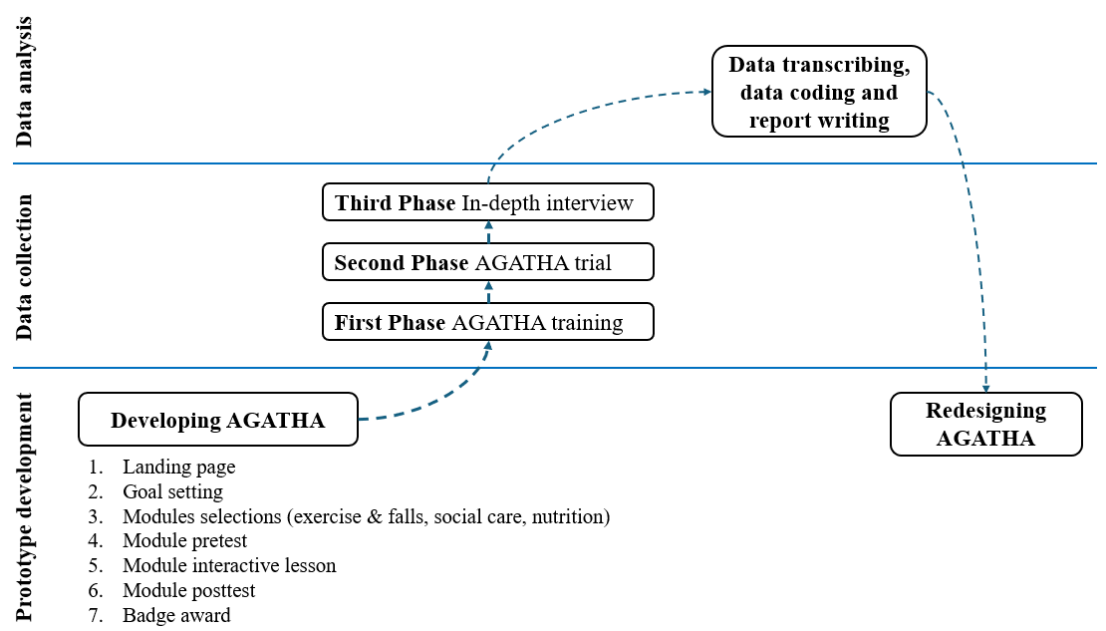
Evaluation workflow and recruitment process of this study. AGATHA: Avatar for Global Access to Technology for Healthy Aging.

The study was conducted in three phases: (1) a training session involving an AGATHA workshop (1 hour); (2) an intervention session, as the AGATHA trial; and (3) an in-depth interview session (90 minutes). In the first phase, participants were introduced to AGATHA, its interface, and functions, and were then provided hands-on training on the digital health coaching program at the Gerontology Laboratory (Figure 1). A research assistant was present to guide participants and clarify any doubts if needed. In the second phase, participants were asked to independently access the program 2 to 3 times a week for at least 15 minutes for up to 2 weeks. Participants who completed the app trial and quiz (see Multimedia Appendix 1) were invited to participate in an in-depth interview. Figure 2 illustrates the evaluation workflow.

A total of 30 in-depth interviews were conducted via face-to-face interviews, phone calls, Zoom, and Google Meet. An interviewer led each interview session with an interview guide (Multimedia Appendix 2) containing open-ended questions in English to ensure the respondents were asked key questions using identical wording. These questions included demographic data, user experiences, challenges, perceptions of AGATHA as a whole and of each module, and perceptions of telemedicine and avatar interaction. The order of questions was changed slightly to allow for fluid conversation flow, and not all questions were explicitly asked if the participants had already answered them. The interviews were conducted by a trained research assistant in the participant’s language of preference and lasted between 45 and 75 minutes. All interviews were audio-recorded and transcribed verbatim for data analysis. Interviews conducted in Chinese were transcribed verbatim and translated into English by a speaker with professional fluency in both Chinese and English. All transcripts were checked for accuracy by the principal investigator. A total of 1622 minutes of interview data were collected. The entire data set consisted of 554 single-spaced pages of text.

After the interview, the researchers read through and coded five transcripts to develop an initial set of themes. The project team then met to review the codes throughout the coding process to reach a consensus on the codes and coding rules process. Discussions were held among project members to refine the codes until a consensus on the themes was reached. An inductive thematic analysis approach, as described by Braun and Clarke [11], was used to analyze the coded data and to develop themes. To support the rigor of the study and its analysis, reflexive reflections of research memos and researcher notes was performed to achieve confirmability of the codes and themes developed through independent analysis, followed by group consensus and discussions about each researcher’s individual perceptions, assumptions, and presuppositions of the data and subsequent analysis. All analyses were performed in NVivo version 12.

### Ethical Considerations

The research protocol was developed by Monash University Malaysia in consultation with the WHO. Study approval was granted by the Monash University Human Research Ethics Committee (project ID 32205 with review reference 2022-32205-74971) on April 19, 2022, and by the Ethics Review Committee of the WHO Regional Office for the Western Pacific (reference number 2022.4.MYS.1.AGE) on May 23, 2022. All participants provided written informed consent and received compensation of RM 800 (approximately US $168) for their participation. All transcripts were deidentified, with a unique ID given to each participant to ensure anonymity.

## Results

### Participant Characteristics

Our sample was gender-balanced, with 15 women and 15 men. Participants in this study were aged between 60 and 78 years. Their sociodemographic characteristics are summarized in Table 1.

**Table 1:** Sociodemographic characteristics of participants who joined in the session (N=30).

Characteristics			Value
Age (years), median (range)			65 (60-78)
**Gender, n (%)**			
	Men	15 (50)	
	Women	15 (50)	
**Ethnic group, n (%)**			
	Malay	9 (30)	
	Chinese	11 (37)	
	Indian	10 (33)	
**Area of residence, n (%)**			
	Perak	10 (33)	
	Selangor	7 (23)	
	Kuala Lumpur	6 (20)	
	Penang	3 (10)	
	Johor	3 (10)	
	Sarawak	1 (3)	
**Urban-rural classification, n (%)**			
	Urban	23 (77)	
	Rural	7 (23)	

### Identified Themes

#### Overview of Main Themes

Based on the thematic analysis of the interview results, the following six themes were identified: (1) prior experience and familiarity with similar digital health coaching programs, (2) perceived benefit, (3) depth of AGATHA health content, (4) suggestions for improvement, (5) user trust in AGATHA, and (6) user interface experience.

#### Theme 1: Prior Experience and Familiarity With Similar Digital Health Coaching Programs

The participants had relatively low familiarity with using smartphones and laptops. They also stated that they actively search the internet for health information such as exercise, social care, and nutrition. However, they had hardly heard of or experienced using digital health coaching programs. They also had minimal exposure to telemedicine.

After retirement during my free time, I use Google and YouTube to search topics on exercise, social care, and nutrition.

W003

Yes, I will seek health information in my WhatsApp group and my friends. They will send me some dietary advice and good food to eat.

W007

Yes, sometimes friends send messages through WhatsApp. They say papaya is one of the best fruit salads and other food that is good. Sometimes I read through advertisements, once in a while on TV. Then in YouTube sometimes when you’re watching the videos in between there are advertisements or companies showing the benefits of the food so in many ways. I do read things that are suitable for me. I take it and what is not I just drop it as I go along the way.

W021

I read somewhere, but I didn’t have time to look into it. At one of our women’s conferences in the church, somebody who was talking about the Senior Health said that we can go into this Digital Health Coach and do some self-health. But I think I honestly didn’t get down to going into it.

W025

I am not comfortable at all. Anything happened. Say your battery run out, you can’t even use it, you know, or say your phone short circuited, the whole thing is wiped out. Anything has happened so I’m not really very confident with all these things, even though I myself am an engineer I still prefer the old way method record it and refer back to my old files and other tasks. All the data that I save, to me it is more reliable and more comfortable.

W008

This is an online screening and electronically speaking which can reach a wide audience rather than a coach in person in a class. It also provides an opportunity for older people to learn how to use electronic digital.

W005

#### Theme 2: Perceived Benefit

Overall, the participants found the digital health coaching program interesting, informative, and beneficial in enhancing their knowledge and awareness about their health, as they could learn valuable and practical information on improving their health. Many participants also felt that using AGATHA would lead them to make positive changes in their health and lifestyle. Some participants expressed their increased motivation to learn more about their health and to be more health-conscious due to AGATHA’s ability to monitor their progress, goal-setting functions, and incentives in the form of badges and certificates.

I think AGATHA is a good app for beginners so if they are not very health conscious, this information will prompt you to start practicing healthy lifestyles.

W003

#### Theme 3: Depth of the AGATHA Health Content

Most participants felt that the health content was informative and easy to understand. However, those with complex illnesses tended to be concerned about the applicability to their specific condition and context. Participants who were more health-literate requested more advanced content (specifically on nutrition and social care). However, others felt that the level of information was adequate and cautioned against increasing the content’s complexity or the topics’ overall length. The participants suggested developing health content they would like to be included in the app. These suggestions consisted of dementias, heart conditions, stroke, mental and spiritual health, nutrition, medication and supplements, COVID-19, physical mobility, safe homes for older individuals, and practical social activity and employment advice.

I think it’s a good course, a spiritual journey. There is nutrition in it, which I like very much, as well as social care courses, which give a lot of inspiration, and sports information is also very good, because I also like sports.

W004

It would be nice to add more. For example, how to take care of the elderly with Alzheimer’s disease, how to take care of the mood of the elderly, especially those who are sick and dying, teach us how to get along with them.

W002

I think I would suggest you put some pictures like fruits because there’s no picture, no different colors. It was quite monotonous. It is very basic.

W009

For me it’s good because the lesson teaches us about what needs to do, what we cannot do. The lesson is good, I like it.

W026

#### Theme 4: Suggestions for Improvement

The first area of improvement suggested by some participants was to provide more opportunities for engagement and interaction such as two-way interactions with the avatar during learning and the opportunities to interact with other participants so that their questions could be posted and the answers shared. Participants also proposed another area of improvement in person-centered care. Suggestions included frequent updates with new content, better access to advanced content, personalized recommendations, and nutrition information more tailored to Malaysia’s cultural and social contexts (eg, using local fruits instead of imported fruits). The third area of improvement put forward by the participants was to increase visual appeal. Participants recommended incorporating fewer text and more pictures, graphics, and animations in various colors to sustain attention and interest. The avatar could also allow for adjustable learning paces. Additionally, some participants suggested incorporating features that enable users to track their progress, such as inputting and viewing their daily vital signs, receiving reminders and prompts to take action, and generating a learning progress report for personal use.

Overall, the app is good, easy to understand. But I am thinking if we have any questions along the way, how are we going to ask AGATHA about the question? Or maybe you all can create another space to let the users put their remarks or any questions.

W006

It was okay actually. Maybe next time she can be designed to my appearance or I can choose a face that I like, you know how they have in all the games you can choose your avatar and sort of design and so maybe it doesn’t have to be the same avatar the whole time. It can keep the name online, but maybe can use someone who was more pleasing to me. But other than that, she was okay.

W014

#### Theme 5: User Trust in AGATHA

Participants had minimal concerns about disclosing personal data with respect to privacy and security due to the minimal information required by AGATHA and limited opportunities for two-way interaction. Given that the content was developed based on WHO technical materials, participants were minimally concerned about the validity of the health content provided and their safety if they applied the advice provided to them. Most participants perceived the information and health content provided to them as valid and trustworthy, which might reflect the quality of the content. However, there were some privacy issues with the website with a warning that it contains potentially unsafe links so that participants had to click “agree and continue.” Thus, it became challenging to convince the older adults of the safety, as some perceived it to be a scam and an insecure website.

No, not at all. I would rather believe let’s say if I have known something and then I get this information from AGATHA, I will prefer to believe AGATHA than my known perspective because what I have known is like hearsay only, no backup.

W006

#### Theme 6: User Interface Experience

Participants perceived AGATHA positively as user-friendly and easy to use, particularly for beginners or less tech-savvy individuals. Examples of the age-friendly attributes in AGATHA were the easy-to-navigate landing page and goal-setting function. Some participants found AGATHA to be occasionally unresponsive and “laggy,” but unanimously concurred that the user interface was best experienced on a laptop or desktop computer and that the interface was difficult to use when accessed on a phone. While the participants could select either the English or Chinese language, they preferred to have more options (eg, Malay and other local languages) for those less fluent in either option presented. Even though the legibility of the written content was not an issue for most participants, visibility posed a challenge due to the smaller font size when viewed on a smartphone. However, the participants appreciated the combination of both written and audio content. Some participants listened to the audio while reading the content for better understanding.

This AGATHA is also very easy to operate and can be used immediately after opening.

W002

Very friendly user, because somebody’s talking to you, explaining to you and making you feel nice. Being a senior citizen like me, I'm not an IT savvy person. So I feel something very interesting.

W021

I think design wise is quite nice because it is segmented, and it is easy enough to use. You don’t really have to know a lot about computers or to know to press a button when it turns up.

W020

I like the AGATHA, the feature because I like to read and I like to listen.

W027

## Discussion

### Principal Results

The findings of this study demonstrated that perceived usefulness, perceived ease of use, interface usability, and the content of this digital health coaching program are among the critical factors in the use of AGATHA for the participants. Most of the study participants understood English, which minimized potential language barriers with using AGATHA. The participants’ interaction with AGATHA provided a proof of concept that digital health coaching programs could be an effective tool for promoting and increasing health literacy and confidence in using digital tools among older adults. The study results also provide insights that may be helpful more broadly in promoting healthy aging among older adults and in developing age-friendly digital tools for this demographic in Malaysia. Given the scarcity of age-friendly digital products and services in Malaysia and other developing economies, it is crucial to ensure that digital technology is tailored to the needs and preferences of older adults and is age-friendly by taking a universal design approach and engaging older adults in the design and development of digital technology.

The participants found AGATHA to be informative and credible. AGATHA provided the participants access to health content and support for self-care, which was perceived as helpful in encouraging them to make health behavior changes [9]. These findings in the Malaysian context align with previous studies conducted in other contexts [12], further supporting the notion that adopting and using digital technology among older adults are active choices.

This study also provides a better understanding of older adults’ preferences and digital adoption behaviors and can thus serve as a guide for how best to cater to their needs for better digital inclusion. As this is a pilot study, future research can extend the study on improving the design by integrating participants’ suggestions. The ability to personalize the program’s contents to cater to specific user needs and profiles would also be a fundamental improvement based on user feedback. For instance, users with particular diseases such as diabetes could receive more targeted information on healthy habits to manage their condition. This could be implemented by asking users about their specific needs and interests, current disease state, medications they are taking, as well as their knowledge level on particular topics upon onboarding. Personalizable user modules or “journeys” could be automatically generated based on these preferences. As some participants struggled to use AGATHA, preferred more languages, and had visibility issues, future developments should be designed to tailor to diverse user competencies, needs, and abilities [13].

The findings of this study also underscore the importance of recognizing older adults as active agents in the digital technology development journey. The insights gained can be extended to other age groups. Therefore, it is important to recognize that older adults should be treated in the same manner as other capable user groups. This resonates with the findings of another study [14] that challenged the notion of older adults as passive recipients or users of digital tools. Developers and designers also need to consider and incorporate older adults’ perspectives, preferences, and unique requirements to ensure that the digital tools created cater to the diverse needs of this population that align with their health and social needs and empower them to lead healthier lives in the digital world.

Overall, the participants did not experience substantial difficulty in using AGATHA. However, they highlighted a few challenges faced during the trial phase. For instance, some participants encountered technical difficulties. The AGATHA app was not ready until 1 week after conducting the three workshops. There were some privacy issues with the website with warnings of unsafe links, and participants had to click “agree and continue.” Thus, it became challenging to convince the older adults of its safety, as some perceived it as a scam and an insecure website. The link to the updated version was not working on one day with a participant. Therefore, immediate feedback was sent to the technical development team and the website was promptly fixed within the day. Some participants opting for the Chinese version had difficulties accessing the app, as the translate function is only available after they log on. Some participants reported that the app response rate was slow, and they had to wait awhile after they clicked “next.” In addition, the app often lagged when they clicked too fast. Participants reported that the log-in process was straightforward, although the “First things first” page, navigation demonstration, and welcome page that appeared each time they logged on distracted them owing to the repetition. They stated that they would prefer to reenter the menu page to continue working on the lessons of interest.

The results of this study concur with similar existing studies on digital health coaching programs for older adults to enhance health knowledge, motivation, and behavior. Castro Sweet et al [15] found significant improvements in self-care and health outcomes among Medicare participants enrolled in a digital health program combined with human coaching. Akin to AGATHA, participants appreciated the motivational aspects and the informative content in enhancing health knowledge and motivation [15]. However, challenges related to technology familiarity, engagement, and program personalization need to be addressed to maximize their effectiveness. Importantly, as highlighted in this study and others, digital health interventions should focus on a user-centered design, accessibility, and personalization to meet the diverse needs of older adults, leveraging technology to promote healthy aging and well-being [16].

### Strengths and Limitations

Our study offers several strengths. First, we used a qualitative approach to gain insights into how older adults engaged with AGATHA. This method provided us with richer insight into how AGATHA can be used to support and promote healthy aging as opposed to using a quantitative survey. Our study also recruited participants from various states in Malaysia and used a purposeful sampling approach to ensure representativeness. Nevertheless, there are some limitations that should be considered. As we did not collect the users’ technology usage information at baseline, we are unclear whether those with better digital literacy were more receptive to the use of AGATHA compared to those with poorer digital literacy. This was highlighted by participants who mentioned that the issues with apps and smart devices are related to their digital knowledge.

### Conclusions

This study found that participants were familiar with technology but had limited experience with digital health coaching programs and telemedicine. Despite this, they found AGATHA to be informative, beneficial for health awareness, and a motivator for adopting healthier lifestyles, particularly praising its goal-setting features and incentives. However, improvements are needed based on the suggestions provided by the older adult participants.

## Appendix

Multimedia Appendix 1 Examples of quiz completion on the app.

Multimedia Appendix 2 Interview topic guide.

## Abbreviations

AGATHA: Avatar for Global Access to Technology for Healthy Aging
ICOPE: Integrated Care for Older People
WHO: World Health Organization

## Data Availability

The data sets generated and analyzed during this study are available from the corresponding author on reasonable request.
