# Correction: Insights Into the Use of a Digital Healthy Aging Coach (AGATHA) for Older Adults From Malaysia: App Engagement, Usability, and Impact Study

**DOI:** 10.2196/60872

**Published:** 2024-05-28

**Authors:** Pei-Lee Teh, Andrei O J Kwok, Wing Loong Cheong, Shaun Lee

**Affiliations:** 1 School of Business Monash University Malaysia Subang Jaya Malaysia; 2 School of Pharmacy Monash University Malaysia Subang Jaya Malaysia

In “Insights Into the Use of a Digital Healthy Aging Coach (AGATHA) for Older Adults From Malaysia: App Engagement, Usability, and Impact Study” (JMIR Form Res 2024;8:e54101), the authors noted an error made in the affiliations of two authors.

The original affiliation was published as:

Pei-Lee Teh^1^, PhD; Andrei O J Kwok^1^, PhD; Wing Loong Cheong^1^, PhD; Shaun Lee^1^, PhD

^1^School of Pharmacy, Monash University Malaysia, Subang Jaya, Malaysia

It will now be changed to:

Pei-Lee Teh^1^, PhD; Andrei O J Kwok^1^, PhD; Wing Loong Cheong^2^, PhD; Shaun Lee^2^, PhD

^1^School of Business, Monash University Malaysia, Subang Jaya, Malaysia

^2^School of Pharmacy, Monash University Malaysia, Subang Jaya, Malaysia

The correction will appear in the online version of the paper on the JMIR Publications website on May 28, 2024, together with the publication of this correction notice. Because this was made after submission to PubMed, PubMed Central, and other full-text repositories, the corrected article has also been resubmitted to those repositories.

