# Supplementary material for: Insights Into the Use of a Digital Healthy Aging Coach (AGATHA) for Older Adults From Malaysia: App Engagement, Usability, and Impact Study
Source: JMIR Form Res. 2024 May 21;8:e54101. doi: 10.2196/54101 (PMC11132589; doi:10.2196/54101)
Supplement: Multimedia Appendix 1 [file formative_v8i1e54101_app1.docx]

## **Appendix 1: Photo of Quiz Completion**

| 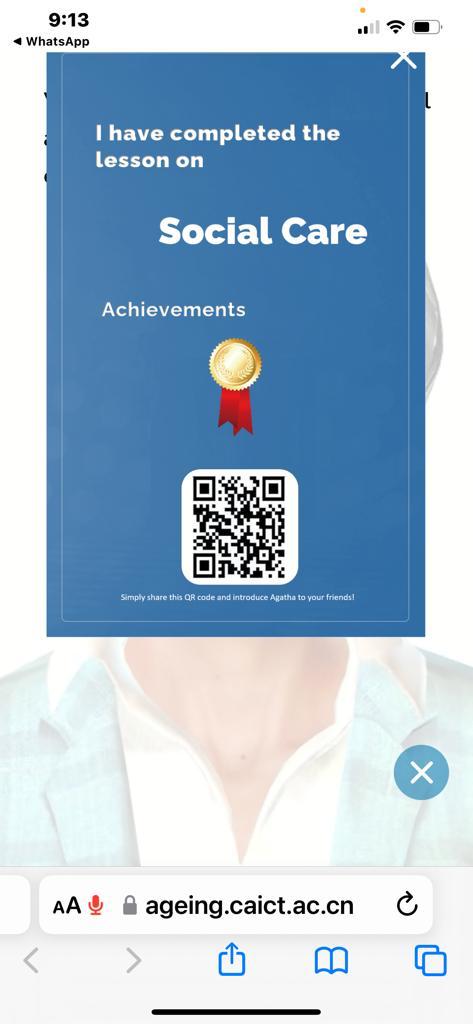 | 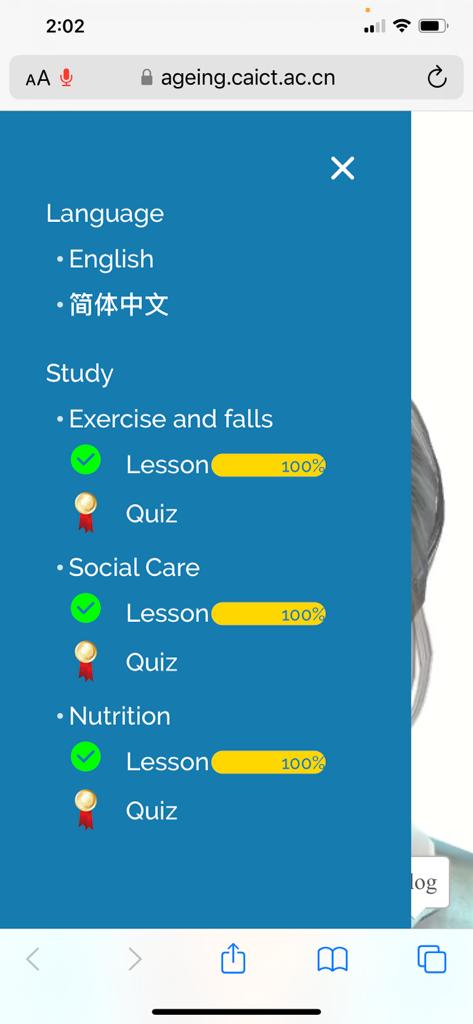 |
| --- | --- |
| Participant W004 completed quiz on Social Care on 14^th^ September 2022 | Participant W003 completed lessons and quizzes on 14^th^ September 2022 |
