# Supplementary material for: Insights Into the Use of a Digital Healthy Aging Coach (AGATHA) for Older Adults From Malaysia: App Engagement, Usability, and Impact Study
Source: JMIR Form Res. 2024 May 21;8:e54101. doi: 10.2196/54101 (PMC11132589; doi:10.2196/54101)
Supplement: Multimedia Appendix 2 [file formative_v8i1e54101_app2.docx]

**Appendix 2: Interview Guide**

INTERVIEW TOPIC GUIDE

Project title: Pilot of AGATHA, A Digital Healthy Ageing Coach for Older People

Start – interviewer introduces the study, explains the purpose of interview, and obtains verbal consent from interviewee.

**Note: This topic guide contains potential questions that can be used during the in-depth and semi-structured interviews held with the study participants. The order and wordings may change slightly to allow conversation flow, and not all questions may be explicitly asked if the participant has already answered them. Some questions and topics may be explored to varying degrees and will be largely guided by the participant.*

| **General Questions** | **Prompts** |
| --- | --- |
| General | Tell me more about yourself (e.g., name, gender, age, employment status, place of residence, household members, ability to use smartphone, what do they use smartphone for, COVID impact on livelihood) |
| Probe on AGATHA feedback | (Assuming all participants have completed the training session on how to use AGATHA)  ● Can you describe how your experience using AGATHA has been like?  ● What other thoughts do you have about the AGATHA app? What do you like and dislike about this app?  ● Do you think you and your friends will use the AGATHA app if it is available? Why yes, or why no?  ● What about the features in AGATHA (e.g., goal-setting, pre-test, lessons, quiz, scoring and badge, progress tracking and voice-interaction)? What did you think was good about the features? Did you find any parts difficult to understand? Is there anything else that we can do to improve the AGATHA? |
| Probe on overall perception | Allow participants to try the AGATHA app.  ● What do you think is a digital health coach?  ● What do you think the AGATHA app is able to help you with?  ● What other health related contents would you want to see on the app?  ● Did AGATHA meet your expectations for a digital health coach? If no, why not?  ● How does AGATHA compare with other digital health coaches you may have tried previously? In what ways were they similar and different? |
| **General Questions** | **Prompts** |
| Probe on the user interface of the AGATHA application | ● Do you find it easy to access and open the app?  ● Is it easy to use? Is it easy to navigate and operate? Do you know where to press for certain functions?  ● What are some of your comments about the visual display? Can you read the texts easily? Is the colour contrast clear?  ● Can you follow the commands/voice commands easily?  ● What do you think of the:  i. Overall appearance  ii. Font style and size  iii. Buttons  iv. Voice, volume, tone, pitch  v. Colour  vi. Speed  vii. Language  viii. Contents and responses |
| Perceptions on healthy ageing information | ● What type of health information would you want to know every day? (e.g., steps count, heart rate, activity level)  ● Do you think you can trust the health information you find on AGATHA? Who do you think provided the information on AGATHA? Would you follow the advice given? Do you have any concerns about the information provided?  ● Do you seek health information regularly? How often? What type of health information?  ● Where do you obtain your health information? Why? (e.g., TV, newspaper, friends)  ● Willingness to revisit if content is updated? How often? |
| Technology and telemedicine | ● What smart tools/gadgets (e.g. smartphone, tablet, laptop, desktop) are you currently using? What do you mainly use them for? What are some issues that you have faced when using these tools and gadgets?  ● Have you received any health consultations through telemedicine before this? If YES, can you tell us what the experience was like? What do you think are the most important factors for a successful telemedicine health consultation?  ● Is somebody helping you use these tools/gadgets? Who? How are they helping you?  ● Have you seen anything like this AGATHA App which I have just showed you, if yes, which one? |
| **General Questions** | **Prompts** |
| Avatar/Digital Health Coach | ● Would you or your friend consider using a digital healthy ageing coach, such as AGATHA? Why or why not?  ● How do you imagine an avatar to look or sound like (e.g., male/female, age, nationality and language/s or dialects spoken, profession, voice-volume, tone, pitch)?  ● Besides asking for health information, what else would you do with the avatar?  ● What do you think can be the limitations and risks of engaging with a digital health coach?  ● What are your suggestions to address these limitations? |
| Usefulness of AGATHA with healthy ageing | ● Would you consider using AGATHA in the future? Why or why not?  ● Do you think AGATHA can help an adult age healthily? Why or why not?  ● Can you think of any friends or family members that would be interested to use this app?  ● How useful did you find AGATHA? What are the current features that are most helpful to you and why?  ● Are there other ways that you find AGATHA useful?  ● Are there any features that you would like AGATHA to have?  ● How can we improve AGATHA to be more user-friendly and accessible so that more ageing adults can use it more easily and frequently?  ● How did AGATHA make you feel? Do you think it will influence how you will behave towards your health and lifestyle? Why or why not?  ● Did you enjoy the interaction with AGATHA? Why or why not?  ● Did you feel safe while using AGATHA? Why or why not?  ● Do you feel empowered meaning more informed and confident in terms of your health decisions? Why or why not? |
| Probe on willingness to pay and perceived feedback | ● How much would you pay for such services?  ● If you can afford AGATHA, would you pay for it? Why or why not?  ● Are you willing to purchase smart devices such as smartwatches to use this app?  ● Knowing it would be free of charge to use AGATHA, would you be willing to use it in the long-term? Would you be willing to recommend it to your family or friends? |
